# Nature, Nurture, and the Meaning of Educational Attainment: Differences by Sex and Socioeconomic Status

**DOI:** 10.1017/thg.2023.6

**Published:** 2023-03-13

**Authors:** Thalida Em Arpawong, Margaret Gatz, Catalina Zavala, Tara L. Gruenewald, Ellen E. Walters, Carol A. Prescott

**Affiliations:** 1 Leonard Davis School of Gerontology, University of Southern California, Los Angeles, California, USA; 2 Department of Psychology, Dornsife School of Letters, Arts and Sciences, University of Southern California, Los Angeles, California, USA; 3 Center for Economic and Social Research, Dornsife School of Letters, Arts and Sciences, University of Southern California, Los Angeles, California, USA; 4 School of Psychology, Chapman University, Orange, California, USA

**Keywords:** Gene-by-environment, sex differences, parental socioeconomic status

## Abstract

Estimated heritability of educational attainment (EA) varies widely, from 23% to 80%, with growing evidence suggesting the degree to which genetic variation contributes to individual differences in EA is highly dependent upon situational factors. We aimed to decompose EA into influences attributable to genetic propensity and to environmental context and their interplay, while considering influences of rearing household economic status (HES) and sex. We use the Project Talent Twin and Sibling Study, drawn from the population-representative cohort of high school students assessed in 1960 and followed through 2014, to ages 68–72. Data from 3552 twins and siblings from 1741 families were analyzed using multilevel regression and multiple group structural equation models. Individuals from less-advantaged backgrounds had lower EA and less variation. Genetic variance accounted for 51% of the total variance, but within women and men, 40% and 58% of the total variance respectively. Men had stable genetic variance on EA across all HES strata, whereas high HES women showed the same level of genetic influence as men, and lower HES women had constrained genetic influence on EA. Unexpectedly, middle HES women showed the largest constraints in genetic influence on EA. Shared family environment appears to make an outsized contribution to greater variability for women in this middle stratum and whether they pursue more EA. Implications are that without considering early life opportunity, genetic studies on education may mischaracterize sex differences because education reflects different degrees of genetic and environmental influences for women and men.

Educational attainment (EA) is used by many researchers as a predictor of important life outcomes, including economic success, health, longevity (Hummer & Lariscy, 2011; Mirowsky, 2017; Montez & Hayward, 2014; Olshansky et al., 2012), and risk for cognitive impairment and dementia (Cook & Fletcher, 2015; Ngandu et al., 2007). These outcomes result from a combination of social, behavioral, and genetic factors (Christensen et al., 2006; Reiss & Neiderhiser, 2000) that also underlie EA. How these factors facilitate getting more education can operate differently by groups, including ones identified by social resources related to socioeconomics or gender (Buchmann & DiPrete, 2006; DiPrete & Buchmann, 2013). Social resources can also modulate the genetic influences on EA (Branigan et al., 2013; Herd et al., 2019), yet the magnitude of modulation is uncertain. Deciphering this will help us to better understand how social factors, or nurture, influence the degree to which genetic factors, or nature, differentially predict EA for different people. Using a twin and family design, we shed light on the extent to which an individual’s educational attainment reflects genetic and environmental influences, and whether the balance of these influences on EA varies for men and women, and disparities in the rearing household’s economic resources.

## Sources of Genetic and Environmental Variance That Influence Educational Attainment

From the biological and genetic perspectives, higher EA stems from some degree of inherited predisposition (Lee et al., 2018; Martin et al., 2011; Okbay et al., 2016). A recent pooled analysis of twin and family studies estimated that 41% of the population variation in EA was due to genetic factors (Silventoinen et al., 2020). An additional 31% and 26% of population variability for EA was attributable to shared and unique environmental factors respectively. Arguably, differences in heritability between groups reflect differences in their environmental variance rather than their genetic variance. For example, one twin study of EA found large differences in relative heritability values by sex in a U.S.-based study in Minnesota (18% for women and 38% for men) but not in Finland (45% for women and 48% for men) (Silventoinen et al., 2004). The bigger difference in heritability between men and women in Minnesota was due to the fact that shared environmental influences on EA were twice as high for women versus men (50% vs. 25%) while in Finland, estimates by gender were similar (42% vs. 37%). Unique variance was also similar for women and men in each location (Minnesota: 32% and 37%; Finland: 13% and 15%). By looking at the contributions of shared environment by sex, it becomes clear that these estimates do not mean that additive genetic influences differ by sex or country, but rather reflect differences in the amount of raw variance explained by the environment. The differences in relative heritability are consistent with the fact that environments related to EA in Finland (i.e., educational policies that emphasize educational equity, social expectations about academic pursuits) are more uniform for males and females (Ahola et al., 2014; Kyrö & Nyyssölä, 2006). For the US, if the amount of raw environmental variance is smaller among men relative to women, and the amount of variance explained by genes is the same in both, then the heritability estimate will be greater for men. This would explain how we might observe an effect where the environment alters the influence of genetics on EA. However, raw variance values are not often provided by studies on EA. Consequently, when proportions of variance (i.e., heritabilities) are compared, it obscures our ability to compare the origins of the group differences.

Additionally, twin models typically assume genetic and environmental components are independent (uncorrelated) and additive. Across generations, social stratification in human populations has resulted in genetic-environment correlations, such as when highly educated parents provide their offspring with both genetic endowment and environmental contexts conducive to higher scholastic success. While we do not have access to parental genetic endowment in our study, we can look at the downstream effects in which such stratification can result in more substantial differences for particular subgroups. This form of stratification exemplifies a gene-by-environment interaction (G × E), where average genetic influence expressed across a population, or heritability, varies by social environment.

## Heritability Differences Associated with Socioeconomic Status (SES)

Heritability-by-SES interactions have been examined most often in twin studies of intellectual abilities. Some studies have reported heritability for cognitive ability to be higher for twins from higher SES compared to twins from lower SES backgrounds (Rowe et al., 1999; Scarr-Salapatek, 1971; Tucker-Drob & Bates, 2016; Turkheimer et al., 2003). This is interpreted to indicate that, on average, high SES environments provide opportunities for individuals to achieve their intellectual potential, whereas individuals from lower SES background have less opportunity for full expression of their genetically driven talents.

Only one prior twin study has tested the heritability-by-SES interaction for EA. The study, conducted in a German sample, found some evidence there was higher heritability among twins whose parents had higher (70–78%) vesus lower (33–40%) educational levels, where such educational levels was used as a surrogate for rearing SES (Baier & Lang, 2019). In addition to not having a direct measure of parental SES, the study assessed EA in a sample aged 22–26, before many individuals had the opportunity to complete their education. Although rearing SES does often include parental EA, the study focused exclusively on transmission of EA from one generation to the next.

## Implications for Sex or Gender Differences in Genetic and Environmental Influences on EA

Average EA has been shown to be higher for men than women in the current population of older aged individuals who are 70+, but we know less about differences between men and women in genetic and environmental sources of variance in EA by their measured social context. As noted previously, heritability differences can under- or overrepresent the average expression of genetic abilities for subgroups of individuals reared in different social environments (Boardman, 2009; Jencks, 1980; Turkheimer & Horn, 2014), including social experiences that differ for men and women. Investigating the basis of sex or gender differences may help explain how these effect relationships between EA and later life health outcomes. For example, if the underlying structure of genetic and environmental influences on EA differs for men and women, such findings could generate hypotheses on why EA predicts cognitive impairment more for men than for women (Gatz et al., 2001; Karp et al., 2004; Letenneur et al., 2000).

To our knowledge, one study has examined sex × environment × genetic interactions for EA and compared estimates for twins raised in very different settings. This study included the aforementioned twins from Finland and Minnesota (Silventoinen et al., 2004). The three-way interaction was also supported by a meta-analysis across 10 countries that reported more of the proportional variance in educational attainment among men was accounted for by genetics, whereas the shared environment accounted for a greater percentage of the variance for women (Branigan et al., 2013). However, in these studies, the reliance on proportions of variance does not facilitate an evaluation of whether nature or nurture is the source of sex differences.

## Current Study

Data for the present study come from the Project Talent Twin and Sibling Study (PTTS), a follow-up cohort from Project Talent, a population-representative study of U.S. high school students first studied in 1960 (Flanagan, 1962), then surveyed again several times via mail and web, between 2014 and 2019 (Prescott et al., 2013; Prescott et al., 2019). Using PTTS for this work provides a rare opportunity to decompose the nature and nurture of EA because measures of family socioeconomic background were collected in 1960 (vs. using retrospective data) from multiple family informants; both twins and siblings are included to provide greater power for decomposing variation (Posthuma et al., 2003) in EA; and EA was collected after individuals had completed formal schooling. PTTS is comprised of individuals born in the early 1940s, and thus expectations of EA differs from contemporary cohorts, in which women born after 1960 are expected to attain more education than men (DiPrete & Buchmann, 2013). However, PTTS is comprised of a sample of today’s 75-year-olds, and evaluating the effects of early life factors on education is highly relevant to current research with older adults, especially when aimed at evaluating sex effects in the relationships between education and later life health outcomes, including cognition and dementia.

Goals of this study are to use a genetically informative design to decipher the etiology and sources of individual differences in educational attainment. We do this by determining the extent to which genetic propensity and shared and unique environmental resources influence educational attainment and whether these factors differ by sex and by family economic resources. Aims are to: (1) investigate how EA differs by sex and socioeconomic background; (2) examine whether there are sex differences in sources of variance underlying EA; and (3) study whether there are sex differences in how genetic influences on EA differ by rearing socioeconomic background. We hypothesize that overall: (h1) men and those of higher socioeconomic background will exhibit more years of EA compared to women and individuals of lower socioeconomic backgrounds; (h2) shared and unique environmental factors will contribute more to individual differences in EA for women compared to men; and (h3) the degree of influence from nature and nurture on EA will differ across groups, with genetic variance being restricted for women and individuals of lower socioeconomic background.

## Materials and Methods

### Subjects

The PTTS (Prescott et al., 2013; Prescott et al., 2019) is a follow-up study to a subsample of PT, which is a population-representative sample of U.S. high school students in 1960, including 377,015 students first tested in 1960 and followed through 1974 (Wise et al., 1979). In 2014, all potential twins and siblings of twins were identified in the original PT study, yielding 5003 eligible participants who were contacted for inclusion in PTTS and for additional follow-up in 2019 on their educational, occupational and health outcomes (Prescott et al., 2019). The final phenotypic sample for the current study includes individuals with complete data on educational attainment and other key variables (i.e., sex, household economic status, twin or sibling status) and includes 3552 individuals from 1778 families (93.6% non-Hispanic White, 3.6% African American, 1.3% Hispanic, and 1.5% Other or Mixed Race). Because we allowed up to two twins and one brother and one sister in extended twin biometric analyses, and some families contained triplets or more than two siblings, six individuals were excluded from these families (details provided in the supplementary material) in the final biometric sample. Thus, the biometric analyses included 3546 individuals from 1778 families, in which 3314 individuals were twins, 197 were non-twin brothers, and 215 were non-twin sisters.

### Measures

#### Years of education attained.

EA reflects the number of years of formal education completed. Years of education was chosen as the outcome as it represents cumulative achievement and helps to determine finer differences with incremental change from underlying influences that achievement levels or number of degrees cannot. We constructed a harmonized EA variable using all available sources, including self- and sibling reports. Among 2532 respondents to the 2014–2019 surveys, EA was available by self-report (*n* = 2304), from responses to surveys conducted 5 and 11 years after expected high school graduation (*n* = 24), or from sibling or cotwin reports in 2014 (*n* = 24). For the 1200 deceased or non-respondents to 2014–2019 surveys, EA was available from sibling or cotwin reports (*n* = 824) or from the 5- and 11-year follow-ups (*n* = 376). EA is coded as: 9 = 9th grade, 10 = 10th grade, 11 = 11th grade, 12 = GED or high school diploma, 14 = associate’s degree, 16 = 4-year degree, 18 = master’s degree, 20 = doctorate. (Validation of sibling reports and other details are in the supplementary material, including Table S1).

#### Household economic status (HES).

Family of origin socioeconomic status was indexed by a composite score formed from student responses on five items on the 1960 Project Talent survey. Item content covered household income, home value and ownership, and presence of furnishings, luxury goods, and books in the household (Wise et al., 1979). (See supplementary material for item wording and response options.) For the 1960 HES composite, first, each student’s response to an item was converted to a *z* score, with a mean of zero and standard deviation of 1, computed by using the raw mean and standard deviation values from all of the 1960 respondents. Next, an individual-level HES score was constructed by calculating the average of each person’s nonmissing *z* scores. Finally, the family HES score was calculated as the average of scores from all participating members in a family. Because preliminary analysis showed that the relation between family HES and EA was not linear, and our interest was in comparing the effect of higher versus lower HES on EA, we constructed three discrete strata based on the distribution of HES calculated in the entire 1960 sample: Individuals scoring in the lowest <25% of the 1960 HES range were designated as *low HES*, those within 25 to <75% of the range were *middle HES*, and those at 75% or higher were *high HES*.

#### Sex and zygosity.

We use the term sex to refer to both biological sex and gender. For all individuals participating in PTTS, the sex provided by their high school in 1960 corresponded to their self-reported gender in 2014. Zygosity refers to the determination of whether twin pairs are monozygotic (MZ, or identical) or dizygotic (DZ, or fraternal). Details on zygosity assignments are provided in the supplementary material.

### Analyses

We conducted individual-level, or phenotypic, regressions to investigate how educational attainment was predicted by rearing-family socioeconomic indicators and whether this differed by sex (raw data plot shown in Figure S1 in the supplementary material). We then fit a series of biometric variance component models to estimate the relative importance of latent genetic and environmental factors and whether these differ by sex and family HES. Data management and individual-level analysis were conducted in SAS v9.4 (SAS Institute, 2018) and biometric variance components analysis of twin and sibling data were completed in Mplus (Muthén & Muthén, 2008).

#### Individual-level analyses.

Individual-level regression models were used to evaluate the effects of sex, HES and the interaction of sex × HES on EA (raw data plot, shown in Figure S2 in the supplementary material). Given the family structure of the data, we fit multilevel regression models with individuals nested within families using PROC MIXED in SAS to obtain unbiased estimates of standard errors. We use explained variance (*R*^2^) between nested models to evaluate the importance of added term(s).

#### Biometric model estimation.

We implemented a univariate twin modeling approach to estimate the sources of individual differences in EA, specified by three estimated genetic and environmental variance components: additive genetic (A), shared environment (S), and unique individual environment (E). When using the ‘ASE’ models, several assumptions are made for each component (Eaves et al., 1978; McArdle & Prescott, 2010). Assumptions are that A represents the effects of genetic alleles that combine additively; that MZ pairs share 100% of additive genetic variance, and on average, DZ pairs and ordinary siblings share 50%; S encompasses all aspects of the environment that they share equally and result in twins being similar, including their rearing environment, family social class, other social factors such as religion, race/ethnicity, as well as effects of community-level factors (e.g., school quality, national policies) (Prescott et al., 2015; Prescott & Kendler, 1995); and E includes all nonshared factors contributing to differences among twins from the same family, such as events uniquely experienced by the individual as well as measurement error. With this design, the variance and covariance of nontwin siblings are modeled in the same way as for a DZ twin (Blokland et al., 2013). Based on the difference in expected genetic resemblance of MZs and DZs, or nontwin siblings, we can construct structural equation models (SEM) to decompose the variance in EA into A, S, and E variance.

Preliminary intrapair correlation calculations (presented in Table S4 in the supplementary material) indicated that EA means, variances and covariances among non-twin sibling pairs (i.e., twin with non-twin sibling) did not differ significantly from those for DZ twin pairs, such that we did not need to include twin environment as a separate component in subsequent model fitting. Calculations for intrapair correlations were conducted with some individuals included more than once, as a co-twin and a sibling of a non-twin sibling. This occurred in 278 families that had a pair of twins and also one non-twin sibling, so that each twin was paired with the co-twin and the sibling; 35 families that had a pair of twins and also two non-twin siblings so that each twin was paired with a co-twin and each sibling; and two families in which there was one twin and two non-twin siblings so the twin was paired with each sibling.

To accommodate the variety of family compositions in number and sex of siblings in our sample, we implemented an expanded twin family modeling approach to allow family structures with up to two twins plus one brother and one sister. Coding details for each family structure are provided in the supplementary methods. We evaluated our hypotheses concerning differences in EA variance sources by fitting a series of sex- and HES-moderated variance components models estimating additive genetic, shared and unique environmental factors using multiple-group SEM. We used a series of five-group SEMs to initially test for sex differences in means and variance structures. We then used a series of 15-group SEMs to partial HES from EA and evaluate models for HES × sex differences, by allowing variance structures to differ by sex and HES levels. Individuals were first assigned to five groups based on sex and zygosity type of the twin pair. Assignment details for family structures with more than two twins or siblings is provided in the supplementary methods. Families in the five groups were further assigned to one of 15 groups, based on rearing family HES (3 strata: low, middle, high) to evaluate HES and HES × sex differences. Models were estimated with full information maximum likelihood (FIML) to minimize the −2 log likelihood (2LL). When comparing alternative models, we used the difference between their −2LL, which is distributed as a *χ*^2^ statistic. Details of using SEM to fit models for twin data are available in greater detail elsewhere (McArdle & Prescott, 2005).

A nonstandard aspect of our analysis approach is the use of HES as a continuous predictor of EA and HES-strata as a categorical moderator of variance components. When a hypothesized moderator is not independent of the outcome, stratifying on it reduces the total variance by removing some of the moderator-outcome covariance. The usual approach for testing moderation of variance components in twin/family models is to estimate simultaneously the biometric structure of the dependent variable, the moderator and their covariance (Neale et al., 2006; Purcell, 2002). This decomposition of the moderator is not necessary in the present case, as HES is by definition a family-level variable and contributes only to shared environmental variance. Variation in EA due to a linear effect of HES is part of the shared (family) variance; any remaining nonlinear effects are included in the estimated latent common variance for the HES strata.

## Results

Table 1 summarizes key variables for the 1876 females (53%) and 1676 males (47%) in the analysis sample with EA quantified in years. Average age at the 2014 follow-up was 69.7 years. Men had significantly higher EA than women (14.2 vs. 13.5 years) and were more likely to graduate college (36.9% vs. 25.9%). HES measured in 1960 did not differ between men and women.

### Differences in Years of Education by Sex and Socioeconomic Background

First, to begin addressing aim 1, we investigate how EA differ by sex and socioeconomic background. In all three strata of HES, we calculated that men had higher mean and greater variability in EA compared to women: low (male *M* = 13.2, *SD* = 2.2; female *M* = 12.6, *SD* = 1.8), middle (male *M* = 14.0, *SD* = 2.5; female *M* = 13.3, *SD* = 2.2), and high (male *M* = 15.6, *SD* = 2.8; female *M* = 14.9, *SD* = 2.5).

Using multilevel regression, we construct a model with EA as the dependent variable that accounts for family relationships, includes fixed effects of sex and 1960 family HES, and adjusts for other indicators of socioeconomic status including father’s education, mother’s education, and father’s occupation. The full model accounted for 20.9% of the variation in EA (details in Table S3 in the supplementary material). Results confirmed hypothesis 1: Compared to females, being male was associated with 0.60 years higher EA. Overall, a 1 *SD* higher HES score predicted 0.82 years more in EA. Adding a sex-by-HES interaction to the model did not increase the explained variance in EA (*R*^2^< .1%) thereby indicating that the observed relationship between EA and HES was the same for men and women.

### Sex Differences in the Underlying Sources of Variance on Education Attained

Second, we address aim 2, whether there are sex differences in the sources of variances underlying EA. First, we calculate pair resemblance by pair sex and relationship type (presented in Table S4 in the supplementary material). We verified that intrapair correlations for EA were higher for MZ (248 female pairs *r* = .69, 196 male pairs *r* = .74) than for DZ pairs (273 female pairs *r* = .56, 240 male pairs *r* = .39), which is evidence for the presence of genetic contributions to individual differences in EA.

**Fig. 1.**
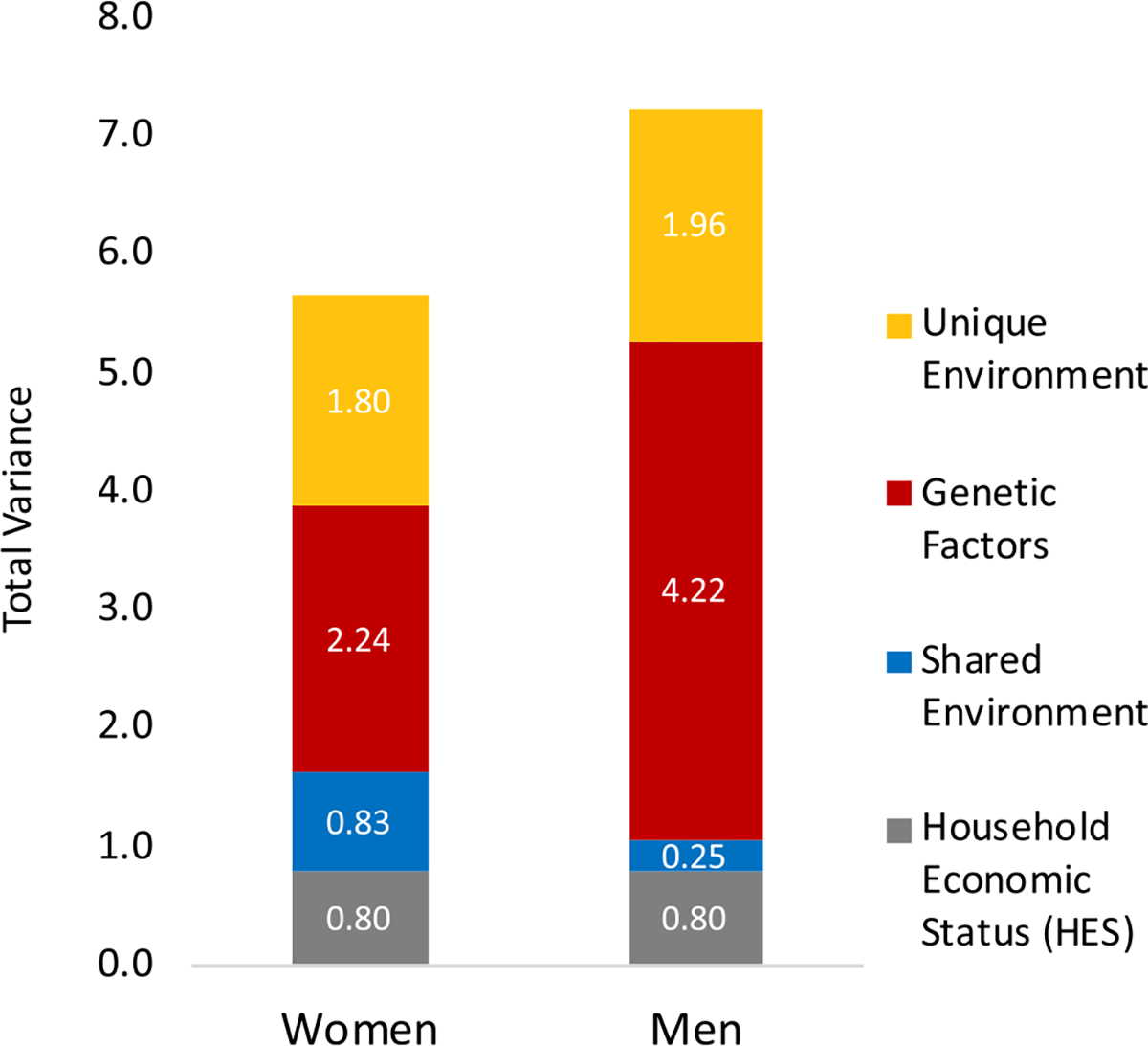
Total variance and components of variance in years of education attained estimated from twin pair relationships. Variance components (y-axis) are divided into sources, from household economic status (HES), shared environment, genetic factors, and unique environment. Columns depict variance sources estimated separately for women (left) and men (right) using an expanded sex-moderation model (sex-specific variance estimates are provided in Supplementary Material, Table S6).

Next, we fit a series of five-group structural equation models (SEMs), in which individuals were assigned to the groups based on sex and zygosity type of the twin pair (MZ females, MZ males, DZ females and female siblings, DZ males and male siblings, opposite-sex DZ twins and siblings) and conducted a general test of equality of variance and components of variance. Model fitting results are presented in Table S5 in the supplementary material. Model-fitting results reject that there is equality across sex (*χ*^2^= 33.59, *df* = 3, model 1 in Table S5 in the supplementary material). The effect of HES contributed equally to EA for men and women, as a test for whether the effect of HES differed across sex showed no difference in model fit (*χ*^2^= 1.35, *df* = 1, model 2 in Table S5 in the supplementary material). Given these results, in all subsequent analyses, variance estimates were allowed to differ by sex, and the HES intercept was equated across sex. Figure 1 depicts how the sexes differ in total variance and magnitudes of genetic and environmental variance for EA, including HES (estimates are provided in Table S6 in the supplementary material). Men had a greater amount of variance overall, and more genetic variance than women. In relative amounts, genetic variance accounted for 51% of the total variance, but within women and men, heritability was calculated as 40% and 58% respectively. Women had more shared environmental variance contributing to EA than men.

#### Moderation of Genetic and Environmental Contributions to Education Attained by Sex and HES

To address aim 3 and the fundamental question of whether education reflects the same influences across different rearing contexts, we evaluated the degree that genetic and environmental influences differ by sex and HES strata. To this end, we fit a series of biometric models using 15-group SEMs. Comparison models were evaluated relative to a baseline model equating variance components across sex and HES strata. All models allowed different intercepts for each sex × HES level. Model-fitting results are presented in Table S7 in the supplementary material. Comparing models, we found that a model allowing variance sources for EA to differ by HES strata as well as sex fit substantially better than the HES-invariance model (*χ*^2^= 70.11, *df* = 12; model 2 in Table S7 in the supplementary material). As shown in Table 2, the total variance in EA increased as HES stratum increased and was greater for men than for women at each stratum.

**Fig. 2.**
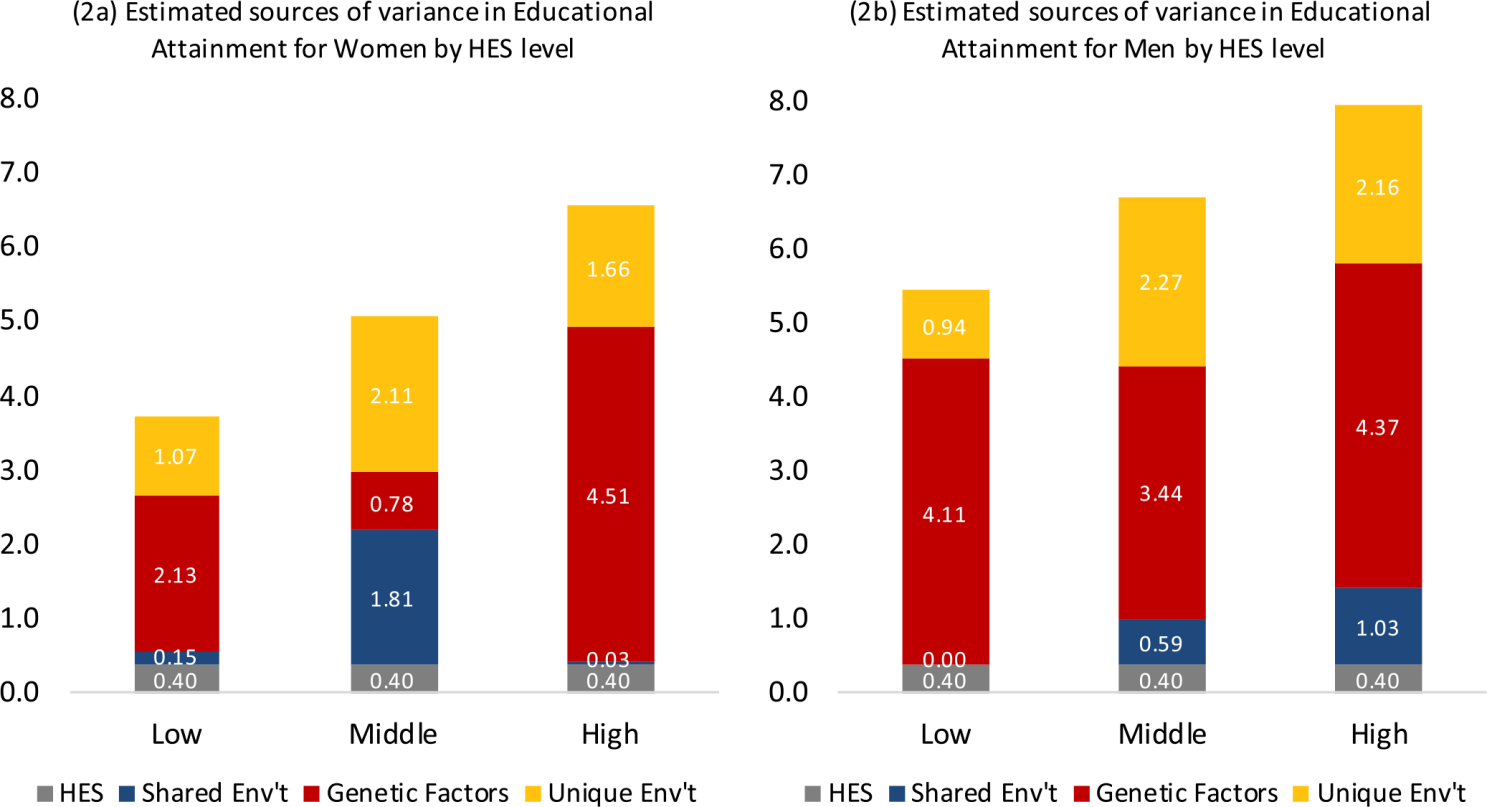
Total variance and magnitude for sources of variance (y-axis) underlying years of educational attainment estimated separately for (a) women and (b) men, by household economic status (HES) strata (x-axis). Columns depict variance sources — from HES, shared environment, genetic factors, and unique environment — estimated separately for each HES strata (model 2 in Supplementary Material, Table S7). Estimates show that HES moderation of genetic variance was present only for women.

What we did not expect, and has not been reported previously, is that HES moderation of genetic variance was present only for women. This is depicted in Figure 2 to emphasize the pattern of variance estimates across HES. A model equating genetic variance across HES for males did not show worse fit than allowing HES-specific genetic estimates (*χ*^2^ = .98, *df* = 2). However, in women, the test for equality of genetic variance across HES was rejected (*χ*^2^= 14.14, *df* = 2). A series of model comparisons showed that underlying differences across HES are due to differences in genetic variance (depicted in Figure 2). Although we hypothesized that genetic variance in EA would be greater with higher strata of HES, this was true for women only, with about double the absolute variance for lowest compared to highest HES. This finding would not have been detected if relying on variance proportions (shown in Table 2) to compare genetic contributions to EA by HES stratum for women. The effect of HES was not linear. For women, genetic factors contribute the most in variance for the high HES stratum and least in variance for the middle HES stratum, with both low and middle HES strata having less variance than the high stratum and less variance compared to men in all strata.

The nonlinear pattern of variance estimates across HES raised the question of how dependent the results were on the cut-points used to define HES strata. We examined this by estimating the sex differences model with seven overlapping subsamples, each comprising 25% of the full sample, defined by a moving window of HES percentile (i.e., 0 to 25, 12.5 to 37.5, 25 to 50, 37.5 to 62.5, 50 to 75, 62.5 to 87.5, and 75 to 100). These estimates, shown in Figure S3 in the supplementary material, illustrate that there were more distinct differences in genetic variance for EA at HES percentile points of 25% and 75% for women, whereas for men differences were more moderate across HES percentiles. This supports a pattern of difference in genetic variance across HES for women and the importance of making comparisons between low, middle and high strata to test for differential contributions of genetic variance.

## Discussion

In this article, we addressed a long-standing question on the importance of nature, via genetic endowment, and nurture, via shared and unique environmental influences, for EA. We found that the balance of nature and nurture underlying EA is not uniform between sexes. First, men and women who were raised in homes with higher household economic status had more years of EA and greater variability in EA than those raised in homes with lower household economic status. Second, we found that overall, there was larger genetic and total variance underlying EA for men than women. Third, nature makes the largest contribution for individuals from the highest family-of-origin economic backgrounds for both men and women. When sex and household economic stratum are both considered, absolute genetic variance contributes similar amounts for men across the economic strata, as well as for women from only the highest economic strata. For women in the lowest and middle economic strata, genetic variance contributes much less to the variability in EA compared to women in the highest economic stratum and to male counterparts across all economic strata. Unexpectedly, for women from the middle economic stratum, it appears that family rearing environment, which may in part reflect parents’ own genetic endowments, may take on an outsized role in contributing to EA. These results confirm that critical interrelationships exist, with nurture moderating effects of nature to alter the range of influence possible on EA. Greater total variance and expression of genetic potential for EA is afforded differently by degree of household economic resources when growing up and in combination with sex or gender.

Findings from this study help us understand the etiology of EA and that EA does not mean the same thing across people, especially for older women today, who were born in the early 1940s. Differences in household economic resources contribute to a disparity in total years of education attained, evidenced by lower overall means and less variability in years of EA for both men and women in the lowest household economic brackets. The finding on differing sources of variance for EA for women by socioeconomic strata was not detectable when examining phenotypic HES × sex effects in predicting EA. This points out that null results in phenotypic models that test sex interactions do not preclude there being differences in etiologies for men and women, particularly with regard to outcomes like EA that are likely influenced by complex processes related to gender socialization and expectations.

Overall, genetic variance accounted for more variation in men’s EA than women’s, at 58% and 40% of the total variance respectively. This is consistent with the ranges for sex-specific calculations reported in prior literature (Baker et al., 1996; Branigan et al., 2013; Heath et al., 1985; Nielsen & Roos, 2015). In turn, the role of nurture was greater for women than for men. This finding supports the interpretation that socio-cultural factors and opportunities shape different trajectories of expression of genetic endowment for men and women (Allan, 2011; Klein et al., 1994). In this cohort, men were able to pursue genetically driven talents for EA irrespective of socioeconomic strata of their families of origin, but women did not have the same benefit unless in the highest HES group. These findings are in line with prior research that has not detected SES-by-sex differences in the heritability of EA (Branigan et al., 2013; Silventoinen et al., 2004) in countries that implement social policies to promote equity in access to educational opportunities (Ahola et al., 2014; Gorard & Smith, 2004; Kyrö & Nyyssölä, 2006). Our findings also support evidence thus far on sex differences by country and birth cohort that show EA likely reflects accumulated genetic sensitivities to the environment (gene-by-SES and gene-by-sex) that are different depending on environmental circumstances (Heath et al., 1985; Silventoinen et al., 2004), and therefore support for G × E effects, by sex and socio-economic group. This points to the results reflecting an opportunity structure and differences in men’s and women’s lived experiences, not biological sex differences.

Shared family estimates from this study are substantially smaller than what is reported in the most recent meta-analysis of proportional variance, yet closer to what is expected given prior knowledge of twin and family studies of other traits (Turkheimer, 2004). Shared environmental variance encompasses family-level resources, including the measured component of household economic status and nonmeasured components, such as family activities and behaviors modeled at home to facilitate exposure to scholarly interests or success in academic pursuits, an living in social and built environments that promote EA. These are not entirely distinguishable from the larger community-level environmental factors that members from the same household share, such as better school quality, or access to healthcare services that promote mental and emotional health. Among women in the middle socioeconomic stratum, the shared family environment appears to make a particularly weighty contribution to greater variability in whether these women pursue higher educational attainment. Conceptually, the family-level resources can have genetic components (e.g., through genetic-environment correlation, effects of assortative mating), but given that twin correlations for both MZ and DZ women were similar and large, this implies that twin and sibling members of the family experience them as a part of their shared, social environment.

Environmental factors on EA that are resources unique to the individual could include parent expectations placed on individual children, peer encouragement, varied learning opportunities offered by teachers, or experiences after school that reinforce scholarly pursuits. When comparing estimates by sex and HES strata, differences in unique, individual-specific experiences are relatively small. Although it is possible these factors have profound influence on particular individuals, findings suggest that adolescents who grew up with more influences from both unique factors and socioeconomic resources in the family are more variable in whether they pursue higher educational opportunities.

The balance of nature and nurture components holds implications for use of EA to predict later life outcomes for different groups. While there are robust and consistent correlations in the literature between education and cognitive function (Opdebeeck et al., 2016; Ritchie & Tucker-Drob, 2018), our prior work showed that genetic variance underlying earlier life cognitive ability overlaps only 11% with genetic variance sources for EA (Arpawong et al., 2018). This finding suggests that the strong relationship between education and cognition is predominantly driven by overlapping nurture components, including life experiences and resources. Relatedly, while education has shown strong predictive ability for cognitive impairment and dementia (Caamaño-Isorna et al., 2006), it has also shown differential ability to predict dementia risk by sex. In particular, education has the lowest predictive value for risk of dementia among women in more impoverished countries (Sharp & Gatz, 2011). Given our findings, we speculate that constrained potential in lower resourced countries and for women means that irrespective of genetic potential, there is less opportunity for these women to attain education; hence, this reduces the overlapping genetic variance between education and cognition. If access to education is driven by within-country environmental factors (e.g., access to resources to pay for education, social prioritization of academic achievement for boys vs. girls), this likely reduces the genetic correlations for EA and cognitive status. In contrast, in higher resourced countries and for men, genetic endowment has more opportunity for expression and thus greater overlap.

In this Project Talent sample, we had limited power to test effects of other social constraints, such as racial/ethnic inequalities. Additionally, we are unable to assess mechanisms by which socioeconomic bracket influences educational differences beyond the variance components quantified, or for those who would not have attended high school given the recruitment design for Project Talent and compulsory schooling laws. Furthermore, we cannot conclude causal associations. For instance, common concerns about causal inference in observational studies center on issues of reverse causation and confounding (McGue et al., 2010). With the present study, we use a longitudinal design where genetics and household economic status precede educational attainment, thus alleviating the first concern. With the second concern, invoking the twin design enables us to control for genetics and shared family environment, and thereby account for the degree of influences from unmeasured environmental factors, or potential confounders (McGue et al., 2010). Thus, although we are not able to establish causality with this study, we are able to make inferences for the direction of effects. A limitation to the design is our inability to make general inferences about siblings because those included are all siblings of twins, and siblings within the same age range of twins, and thus are not representative of the experience of all siblings within families. Lastly, results are likely cohort specific because our estimates align well with prior research evaluating variance sources in education in individuals born between 1940 and 1961 (Heath et al., 1985). Follow-up analyses in younger cohorts will be important to compare differences in findings.

## Supplementary Material

Supplementary Material

## Figures and Tables

**Table 1.** Descriptive information by sex on 3552 twins and siblings from 1778 families

	Women	Men		
	(*n* = 1876)	(*n* = 1676)	Difference between women and men	
	*M* (*SD*)	*M* (*SD*)	Test statistic	Cohen’s *d* (CI)

Age at 2014 follow-up (y)	69.7 (1.2)	69.8 (1.2)	t=−2.14*	−0.07 (−0.14, −0.006)

1960 Household Economic Status (HES) Index	3.2 (0.7)	3.2 (0.7)	t=0.53	0.02 (−0.05, 0.08)

Educational attainment (y)	13.5 (2.4)	14.2 (2.8)	t=−8.18***	−0.27 (−0.34, −0.21)

College graduate (%)	25.9	36.9	*X*^2^ =49.67***	−

Note: HES variables were scaled in *z* scores for analysis though converted to raw scores in this table to be interpreted as the range of 0 (low) to 5 (high), with high HES indicating more household resources. Range for age was 67 to 72 and for educational attainment was 9 to 20 years. M, mean; SD, standard deviation; *CI,* confidence interval.

* *p* < .05

*** *p* < .001.

**Table 2.** Genetic and environmental sources of variation in educational attainment by family rearing Household Economic Status (HES) level, separately for females and males

	Low HES			Middle HES			High HES		
A. Source of variance for females	Var	SE	%	Var	*SE*	%	Var	SE	%

Additive genetic	2.13	0.30	56.8	0.78	0.29	15.4	4.51	0.27	68.3

Unique environment	1.07	0.12	28.6	2.11	0.11	41.3	1.66	0.14	25.1

Shared environment	0.15	0.22	4.0	1.81	0.24	35.5	0.03	0.08	0.5

Family HES	0.40	0.07	10.7	0.40	0.07	7.9	0.40	0.07	6.1

Total variance	3.75	-	100.0	5.10	-	100.0	6.59	-	100.0

	**Low HES**			**Middle HES**			**High HES**		
**B. Source of variance for males**	**Var**	**SE**	**%**	**Var**	**SE**	**%**	**Var**	**SE**	**%**

Additive genetic	4.11	0.24	75.5	3.44	0.61	51.3	4.37	0.69	54.9

Unique environment	0.94	0.11	17.2	2.27	0.14	33.8	2.16	0.19	27.1

Shared environment	0.00	0.00	0.0	0.59	0.19	8.8	1.03	0.62	13.0

Family HES	0.40	0.07	7.4	0.40	0.07	6.0	0.40	0.07	5.0

Total variance	5.45	-	100.0	6.71	-	100.0	7.97	-	100.0

Note: Var, variance; *SE*, standard error; %, percent of total variance.

HES levels are based on quartiles from the full 1960 Project Talent sample: Low HES includes 0 to 25 percentile; Middle is 25 to 75 percentile; High is above 75 percentile.

Variance estimates are from a model with intercepts and variances allowed to vary by sex and each level of HES (Model 2 in Supplement Table S7). Family HES is estimated across all strata and equated over sex.

## Data Availability

Data used for this study is available for approved researchers: https://www.air.org/project/project-talent
