# Supplementary material for: Nature, Nurture, and the Meaning of Educational Attainment: Differences by Sex and Socioeconomic Status
Source: Twin Res Hum Genet. Author manuscript; Available in PMC 2023 Sep 28. (PMC10497722; doi:10.1017/thg.2023.6)
Supplement: Supplementary Material [file NIHMS1906318-supplement-Supplementary_Material.docx]

*Twin Research and Human Genetics*

Nature, Nurture and the Meaning of Educational Attainment: Differences by Sex and Socioeconomic Status

Authors: Thalida Em Arpawong, Margaret Gatz, Catalina Zavala, Tara L. Gruenewald, Ellen E. Walters, Carol A. Prescott

**Supplementary Material file includes:**

Supplementary text

Figures S1 to S3

Tables S1 to S7

SI References

**Supplementary Methods**

***Sample***

*Family structure.* In accommodating the variety of family compositions in number and sex of siblings, we implemented an extended twin family model to allow family structures with up to two twins plus two siblings that included up to one brother and one sister. As a result, 6 individuals in the phenotypic analysis sample were excluded from the biometric analyses, as follows: In 5 families with more than two siblings, two were randomly selected for inclusion. For 1 twin pair with unknown zygosity, one twin was randomly selected for inclusion in the analyses. Of the 13 triplet sets, complete data was available in 4 sets. In 3 sets, the MZ pair was retained and the DZ member was recoded as a sibling. In 1 set of 3 MZ females, 1 was randomly excluded from the analysis. This resulted in family structures in which 1061 families had a pair of twins only, 278 families had a pair of twins and one non-twin sibling, 35 families had a pair of twins and two non-twin siblings, 402 families had a pair comprised of a twin and non-twin sibling, and two families that had one twin and two non-twin siblings.

***Measures***

*Years of education attained (EA).* The variable for educational attainment reflects the estimated number of years of formal education completed. At baseline, all participants were in high school in 1960, and participated in follow-up surveys at various timepoints through 2019.

Respondents were assigned the maximum of their recent self-report values from the 2014-2019 surveys. If the respondent did not have a recent self-report value, they were assigned the maximum of their self-report from the 1965-1974 surveys and any sibling reports from the 2014 and 2019 surveys. All available data including age when last attended school, year and month of birth and grade in 1960 were used to refine reports of less than a high school degree and assign a level of high school grade completion where possible. Because Project TALENT data were collected in high school classrooms, no participant had less than 9 years of education. Response options to the educational attainment item varied across surveys. Item categories were harmonized using a rational approach to create a common 8-level scale for years of education attained as follows: 9=9^th^ grade, 10=10^th^ grade, 11=11^th^ grade, 12=GED or high school diploma, 14=Associate’s degree, 16=4-year degree, 18=Master’s degree, 20=Doctorate.

Agreement between self and sibling reports of educational attainment were high, with pairwise correlations ranging from r=.78 between dizygotic opposite sex pairs to r=.87 between monozygotic female twin pairs (SI Appendix, Table S1). Reports among all non-twin sibling-sibling pairs ranged from r=.83 to .85.

*Family socioeconomic status (SES).* A household economic status (HES) index based on five items from the 1960 Project Talent inventory(1) was created. (See below for wording and response options). Three additional items assessing father’s education, mother’s education and father’s occupation (also described below), were employed as separate markers of SES.

*Items in the household economic status (HES) index.* Five items were used to create the HES index. The first item was, “If your family has bought (or is buying) your home, what is its present value?”. Five response options were given ranging from “Under $6,000” to “More than $22,000”, with a sixth option for “We are renting our home.” The second item was, “Please make the best estimate you can of your family’s total income for the last year (1959).” Five response options were given ranging from “Less than $3,000” to “$12,000 or more”, with a sixth option for “I can’t estimate this.” The third item was, “How many books are in your home?”. Six response options were provided, ranging from “Non, or very few (0-10)” to “A room full – a library (501 or more).” The fourth and fifth item were prompted by the question “How many of the following articles are in your home?” with the fourth item listing, “Automatic washer, automatic clothes dryer, electric dishwasher, electric or gas refrigerator, vacuum cleaner, home food freezer (separate from refrigerator)”. Six response options were provided ranging from “None” to “Five or six”. The fifth item asked about, “Telephone, television set, radio, phonograph” and provided five response options ranging from “None” to “Four”. Responses to these items were provided by individuals, then an individual-level HES score was constructed by calculating the average of each person’s non-missing z-scores. Finally, the family HES score used for analysis was calculated as the average of scores from all participating members in a family.

*Mother’s and father’s educational level*. Educational achievement was reported by respondents for each parent on a scale of 1 (none, or some grade school) to 10 (completed doctorate or professional degree) with a value of 4 representing graduation from high school(1). We took the highest level of education from each parent to represent parents’ educational level, calculated a standardized z-score for each individual, then took the mean score from all individuals in a family to represent the family mean for parents’ education.

*Father’s occupation*. Type of work reported for the father was initially assessed as 17 occupation types. These were coded by Project Talent researchers into levels ranging from 1 (farm or ranch worker, workman or laborer, private household worker) to 5 (official, professional)(1). We calculated the mean level across all individuals in a family to represent father’s occupation.

*Grade in 1960.* High school grade level in 1960.

*Zygosity.* The method for assigning zygosity in the PTTS sample is described extensively elsewhere(2-4). Multiples were identified from the Project Talent Base Year data, assessed in 1960(4). Next, confirmed pairs were assigned to be monozygotic (MZ, or identical) or dizygotic (DZ, or fraternal) twins using an assignment algorithm and case reviews when necessary. We summarize the procedures here. First, an algorithm was adapted from Benyamin, Deary and Visscher(5) and applied to 2014 survey responses from the respondent, cotwin and available siblings. Items asked about: the physical similarity of the twins; whether their identities were confused by others; similarity when young adults of their hair color and texture, eye color and facial features; and their opinion on being monozygotic (MZ, or identical) or dizygotic (DZ, or fraternal) twins. These items have been shown to achieve 90-95% accuracy for zygosity assignments(6-8). The algorithm was applied to the responses of each person (twin or sibling) who reported on the similarity of a twin pair. If there were inconsistencies among the raters’ scores, the pair was referred for further case-by-case review by the investigators. No DZ opposite sex pairs required further review. Same-sex MZ and DZ pairs (twins and all triplets) were reviewed using all available information to rate similarity, including twin responses to the 2014 survey items, text responses to open-ended items, sibling responses, high school yearbook photos, and 1960 reports on height and weight(4). Sibling agreement was high (Table S1). Then, a consensus zygosity was assigned. Of the 2253 pairs, 588 were assigned as MZ, 1346 were DZ, and 319 were unassigned zygosity (UZ) because it could not be determined from the available information.

**Supplementary Results**

As shown in Table S1, agreement between self and sibling reports of educational attainment were high, validating the use of sibling reports for deceased and non-responding individuals.

As shown in Table S2, associations between EA and 1960 family socioeconomic status indicators were similar for females and males.

In initial stepwise regression models shown in Table S3, we evaluated a composite 8-item index of family SES in 1960 and individual family SES indicators as four separate predictors of EA: father’s education (1 item), mother’s education (1 item), father’s occupation (1 item), and household economic status (5 items). All models were adjusted for grade level in 1960 and gender. Although grade did not have a significant effect on EA, it was retained as a covariate to adjust for age in 1960 and the possibility that the sample is more selective for those who were in higher grade levels in 1960, a group who were less likely to drop out of school earlier in high school.

When adding the SES composite score to the base model, it accounted for 17.9% of the variation in EA. In comparison, the four separate SES predictors accounted for 18.7% of the variation and was more informative for partialling effects of SES on EA. The regression model with just the SES composite score showed that a 1 SD higher SES composite index predicted 0.12 years higher EA. In contrast, when separating the SES components, 1 SD higher score for mother’s or father’s education predicted 0.33 to 0.34 years higher EA whereas a 1 SD higher score for HES predicted 0.81 years higher EA. For all subsequent models, we chose the single indicator of HES to represent family socioeconomic status for several reasons. Primarily, we were not focused on assessing transmission of EA from one generation to the next, but rather focused on the effect of economic status experienced by the family. Also phenotypically, HES showed a stronger effect on EA (*b*=0.81, SE=0.10) than father’s education (*b*=0.34, SE=0.06), mother’s education (*b*=0.33, SE=0.06), or father’s occupation (*b*=0.38, SE=0.06). Lastly, HES accounted for the majority of variation in EA (12.0% of 18.7% accounted for by the four SES indicators) when included in the model with father’s education, mother’s education, and father’s occupation (which accounted for 6.7% together). Thus, main analytical models excluded covariates of either parents’ education and father’s occupation to better evaluate effects of the family’s economic status on EA.

Table S4 shows how twin and sibling pairs differ in the underlying influences on EA from evaluating variances and twin and sibling pair resemblance, by pair sex and relationship type. Group means and correlations (SI Table S4) did not differ substantially when comparing DZ twins to siblings, indicating little evidence for a special twin environment (such as prenatal or age-specific postnatal factors).

Table S5 shows that from model fitting results for alternative variance structures for EA, structures differed substantially by sex and HES.

Table S6 shows the estimates for each variance source, in raw values and as a proportion of total variance for that subgroup, from a model that allowed the means, intercepts, and variances in EA to differ by sex.

Table S7 shows that from model fitting results, a model equating genetic variance across HES for males did not show worse fit than allowing HES-specific genetic estimates whereas in females, the test for equality of genetic variance across HES was rejected.

Interpretation of biometric estimates relies on a number of model assumptions, including random mating of parents for the characteristic being studied and the common environment being the same for MZ and DZ twin types. Spousal resemblance for EA exists, but the impact of this on variance estimates depends on the degree to which resemblance is due to direct or indirect selection. These assumptions are not testable in the present case so we evaluate the impact of possible assumption violations when we discuss model results.

**Supplementary Discussion**

***Twin-Sibling Design in Project Talent***

Design of the Project Talent Twin and Sibling (PTTS) study sample used for this study allows us to test assumptions of the twin model and to better understand sources of environmental effects. Strengths of the current study design and our findings are that we include twins and siblings from the same families, which enables a more precise assessment of rearing environment when estimating genetic influences (e.g., rearing family environment was reported on by more than one family member). Another strength of using the PTTS sample for this work is that it was drawn from a population-representative sample of high school students who were assessed during adolescence for early life environment. This frees us from the bias of retrospective recall in characterizing family SES. Reliability of family SES was further strengthened by using reports from more than one family member. Moreover, respondents reported on their own educational attainment as well as the educational attainment of their twins and siblings, offering us the ability to assess accuracy in self-report.

The larger goal of the present study is to expand our understanding of how genetic influences on education differ by gender and socioeconomic level. The large sample size of twins and siblings in PTTS provides the statistical power for testing sex and HES effects.

**Supplementary Table S1**. Inter-Rater Agreement Between Respondent and Sibling Reports of Respondent Educational Attainment

|  | Female-Female | Male-Male | Female-Male |
| --- | --- | --- | --- |
| Respondent with Identical (MZ) Cotwin | 0.86 | 0.83 | -- |
| Respondent with Fraternal (DZ) Cotwin | 0.80 | 0.85 | 0.76 |
| Respondent with Non-twin Sibling | 0.85 | 0.80 | 0.81 |

Note. Values are pairwise Spearman correlations based on years of education attained (EA) variable.

**Supplementary Table S2**. Correlations Among 1960 Family Socioeconomic Status (SES) Variables and Respondent Years of Education Attained, Separately for Men and Women

|  | Family SES Composite | Father’s Education | Mother’s Education | Father’s Occupation | HES Index | Educational Attainment |
| --- | --- | --- | --- | --- | --- | --- |
| 1960 Family SES Composite | -- | 0.60 | 0.56 | 0.51 | 0.81 | 0.40 |
| Father’s Education | 0.61 | -- | 0.57 | 0.41 | 0.46 | 0.33 |
| Mother’s Education | 0.58 | 0.54 | -- | 0.34 | 0.40 | 0.30 |
| Father’s Occupation | 0.59 | 0.47 | 0.37 | -- | 0.38 | 0.31 |
| Household Economic Status (HES) Index | 0.82 | 0.47 | 0.41 | 0.44 | -- | 0.35 |
| Respondent Educational Attainment | 0.40 | 0.30 | 0.30 | 0.32 | 0.36 | -- |

Correlations for females are below diagonal; for males above diagonal. Values are Pearson correlations. N’s vary due to incomplete data. Female N’s: 1,758 to 1,876; male N’s: 1,554 to 1,676.

**Supplementary Table S3**. Results from Individual-Level Regression Analyses Predicting Years of Education Attained from 1960 Family Socioeconomic Status (SES) and Sub-indices

|  | Model 0: Baseline | Model 1:  SES composite | Model 2:  SES indices | Model 3:  sex*HES |
| --- | --- | --- | --- | --- |
|  | Est (SE) | Est (SE) | Est (SE) | Est (SE) |
| Intercept | 14.13 (0.06)^***^ | 14.15 (0.05)^***^ | 14.15 (0.05)^***^ | 14.15 (0.05)^***^ |
| Sex (Female=0, Male=1) | 0.60 (0.08)^***^ | 0.61 (0.08)^***^ | 0.60 (0.08)^***^ | 0.60 (0.08)^***^ |
| 1960 Grade level | 0.02 (0.04) | -0.03 (0.04) | -0.0001 (0.04) | -0.003 (0.04) |
| *1960 Family SES* |  |  |  |  |
| SES composite index | -- | 0.12 (0.005)^***^ | -- | -- |
| Father’s education | -- | -- | 0.40 (0.07)^***^ | 0.40 (0.07)^***^ |
| Mother’s education | -- | -- | 0.32 (0.06)^***^ | 0.32 (0.06)^***^ |
| Father’s occupation | -- | -- | 0.36 (0.06)^***^ | 0.36 (0.06)^***^ |
| Household economic status (HES) | -- | -- | 0.82 (0.10)^***^ | 0.82 (0.10)^***^ |
| *Interaction with sex* |  |  |  |  |
| Sex x HES | -- | -- | -- | 0.24 (0.14)^#^ |
| *R^2^* | 1.48% | 19.96% | 20.94% | 20.39% |
| $\boldsymbol{\Delta}$*R^2^* | -- | 18.48% | 19.47% | 0.04% |

Notes.

These phenotypic regression models include 3,552 twins and siblings from 1,778 families.

#p-value<0.10; ***p-value<0.001. Based on females and males with data on all predictors. Standard errors are adjusted for multiple people per family. $\boldsymbol{\Delta}$R^2^ for models 1 and 2 are calculated in comparison to model 0, the baseline model. $\boldsymbol{\Delta}$R^2^ for model 3 is calculated in comparison to model 2.

**Supplementary Table S4**. Pairwise Resemblance for Years of Education Attained Among Twin and Sibling Pairs

|  | Monozygotic Twin Pairs | Dizygotic Twin Pairs | Sibling Pairs |
| --- | --- | --- | --- |
| *Female Pairs* | | | |
| N_p_ | 248 | 273 | 223 |
| Mean (SD) | 14.0 (2.4) | 13.9 (2.5) | 13.8 (2.4) |
| r (SE) | 0.69 (0.05) | 0.56 (0.05) | 0.39 (0.06) |
| *Male Pairs* | | | |
| N_p_ | 196 | 240 | 163 |
| Mean (SD) | 15.1 (2.8) | 14.4 (2.8) | 14.5 (2.7) |
| r (SE) | 0.74 (0.10) | 0.39 (0.07) | 0.50 (0.07) |
| *Female - Male Pairs* | | | |
| N_p_ | -- | 418 | 385 |
| Mean (SD) | -- | 13.9 (2.4) | 14.0 (2.4) |
| r (SE) | -- | 0.41 (0.04) | 0.39 (0.05) |

Notes.

N_p_ = number of pairs; r = Pearson correlation coefficient; SD = standard deviation; SE = standard error. Sibling pairs group combines twin-sibling and sibling-sibling pairs. Some individuals contribute to more than one pair type, with a total of 2111 complete twin-twin, twin-sibling, or sibling-sibling pairs across families. In subsequent analyses and variance components estimation, each individual is included only once.

**Supplementary Table S5**. Five-Group Model Fitting Results for Alternative Variance Structures for Educational Attainment, Associated with Sex and Household Economic Status (HES)

| **Model for Variance Structure** | | χ^2^ | **df** | **Comparison Model** | $\boldsymbol{\Delta}$χ^2^ | $\boldsymbol{\Delta}$**df** | **Interpretation** |
| --- | --- | --- | --- | --- | --- | --- | --- |
| 0 | Sex= | 153.16 | 92 | -- | -- | -- | -- |
| 1 | Sexes differ in ACE | 119.57 | 89 | 0 | 33.59 | 3 | Different variance structures for males and females |
| 2 | Allow bHES to vary for Males & Females | 118.22 | 88 | 1 | 1.35 | 1 | Effect of HES (bHES) is invariant over sex |

Notes.

All models allow intercept differences by sex, based on 3,546 twins and siblings from 1,778 families

χ^2^ is the chi-square test statistic, $\Delta$χ^2^ is the difference in chi-square relative to the comparison model

df = degrees of freedom, $\Delta$df is difference in degrees of freedom relative to comparison model

ACE refers to the Additive genetic, Common environment, and unique Environment variance components

**Supplementary Table S6**. Estimated Genetic and Environmental Contributions to Individual Differences in Educational Attainment, from the Sex-Moderated Model (M1) with Household Economic Status (HES) as a covariate

| **Source of Variance** | **Females** | | | **Males** | | |
| --- | --- | --- | --- | --- | --- | --- |
|  | **Variance** | **Standard Error** | **%** | **Variance** | **Standard Error** | **%** |
| Additive Genetic | 2.24 | 0.23 | 39.5 | 4.22 | 0.20 | 58.4 |
| Unique Environment | 1.80 | 0.08 | 31.8 | 1.96 | 0.09 | 27.1 |
| Shared Environment | 0.83 | 0.19 | 14.6 | 0.25 | 0.14 | 3.4 |
| Family HES | 0.80 | 0.04 | 14.1 | 0.80 | 0.04 | 11.1 |
| Total Variance | 5.67 | -- | 100.0 | 7.23 | -- | 100.0 |

Notes.

Variance estimates are from a model with intercepts and variances allowed to vary by sex and family HES is estimated across range of HES and equated over sex (Model 1 in SI table S5).

**Supplementary Table S7**. Fifteen-Group Model Fitting Results and Interpretations of Comparing Tests for Alternative Variance Structures for Years of Education Attained, by Sex and Household Economic Status (HES)

| **Model for Variance Structure** | | χ^2^ | **df** | **Comp- arison Model** | $\boldsymbol{\Delta}$χ^2^ | $\boldsymbol{\Delta}$**df** | **Interpretation** |
| --- | --- | --- | --- | --- | --- | --- | --- |
| 0 | Sex=, HES= | 674.62 | 280 | -- | -- | -- | -- |
| 1 | ACE varies by sex, HES = | 638.89 | 277 | 0 | 35.73 | 3 | Sex differences in variance structure |
| 2 | ACE varies by sex & HES | 568.78 | 265 | 1 | 70.11 | 12 | Structure is not invariant across HES after accounting for sex differences |
| 3M | Male A equated across HES | 569.76 | 267 | 2 | 0.98 | 2 | For Males: Genetic variance does not differ across HES level |
| 3F | Female A equated across HES | 582.92 | 267 | 2 | 14.14 | 2 | For Females: Genetic variance does differ across HES level |

Notes.

All models fit with HES intercept equated over sex within HES level, and HES variance and beta weight is equated across 15 groups. Model is based on 3,546 twins and siblings from 1,778 families.

Model 1 evaluates hypotheses about sex differences, with the fit compared to the baseline model 0.

Model 2 evaluates differences in variance components associated with HES after accounting for sex differences, compared to model 1.

Model 3M evaluates differences in male genetic variance associated with HES, compared to model 2.

Model 3F evaluates differences in female genetic variance associated with HES, compared to model 2.

Models 3M and 3F were configured for estimates constrained to be positive.

χ^2^ is the chi-square test statistic, $\Delta$χ^2^ is the difference in chi-square relative to the comparison model

df = degrees of freedom, $\Delta$df is difference in degrees of freedom relative to comparison model

ACE refers to the Additive genetic, Common environment, and unique Environment variance components


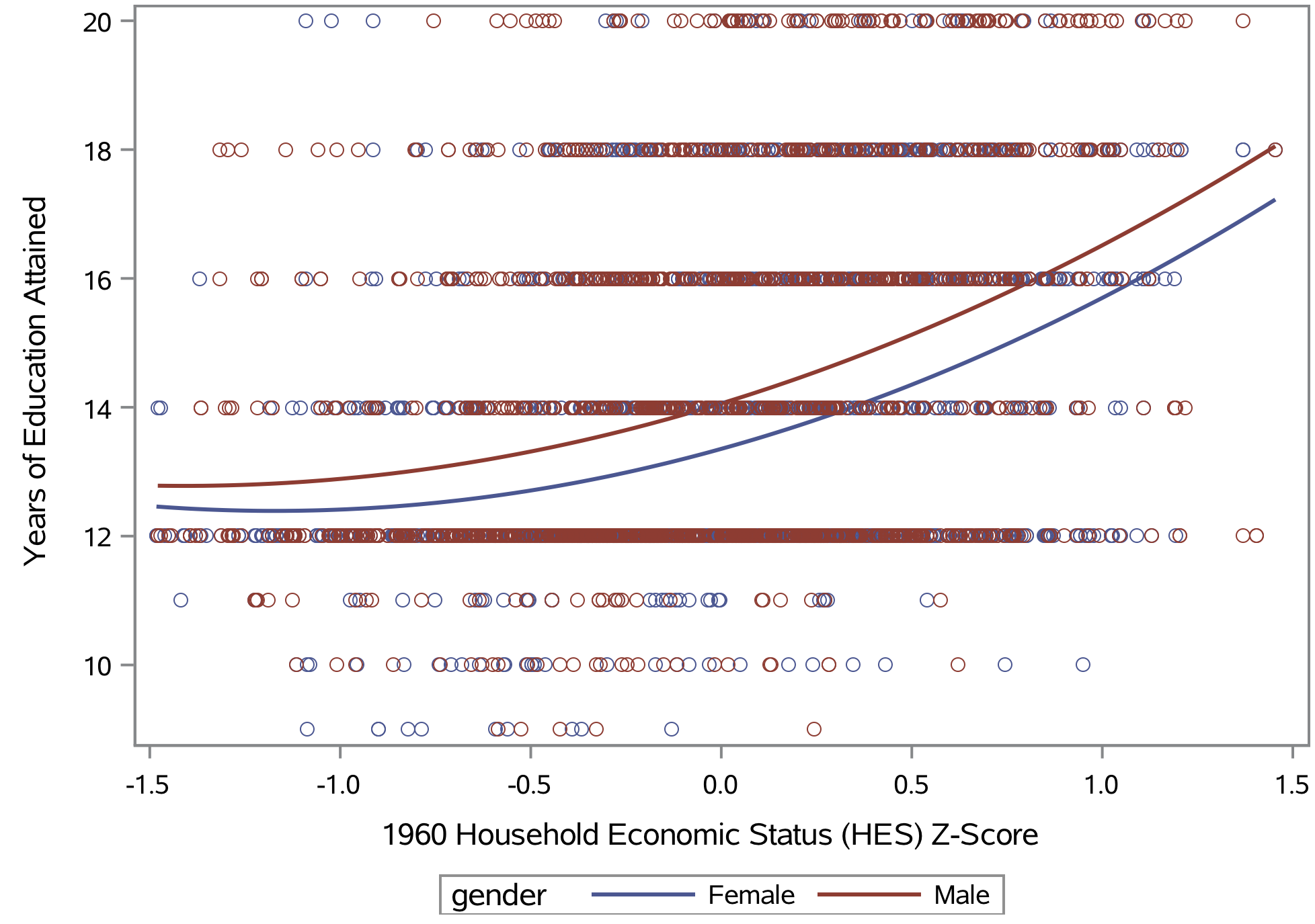


**Supplementary Figure S1**. Data plot for 1960 family rearing household economic status (HES; x-axis)and years of education attained (y-axis), separately for females and males


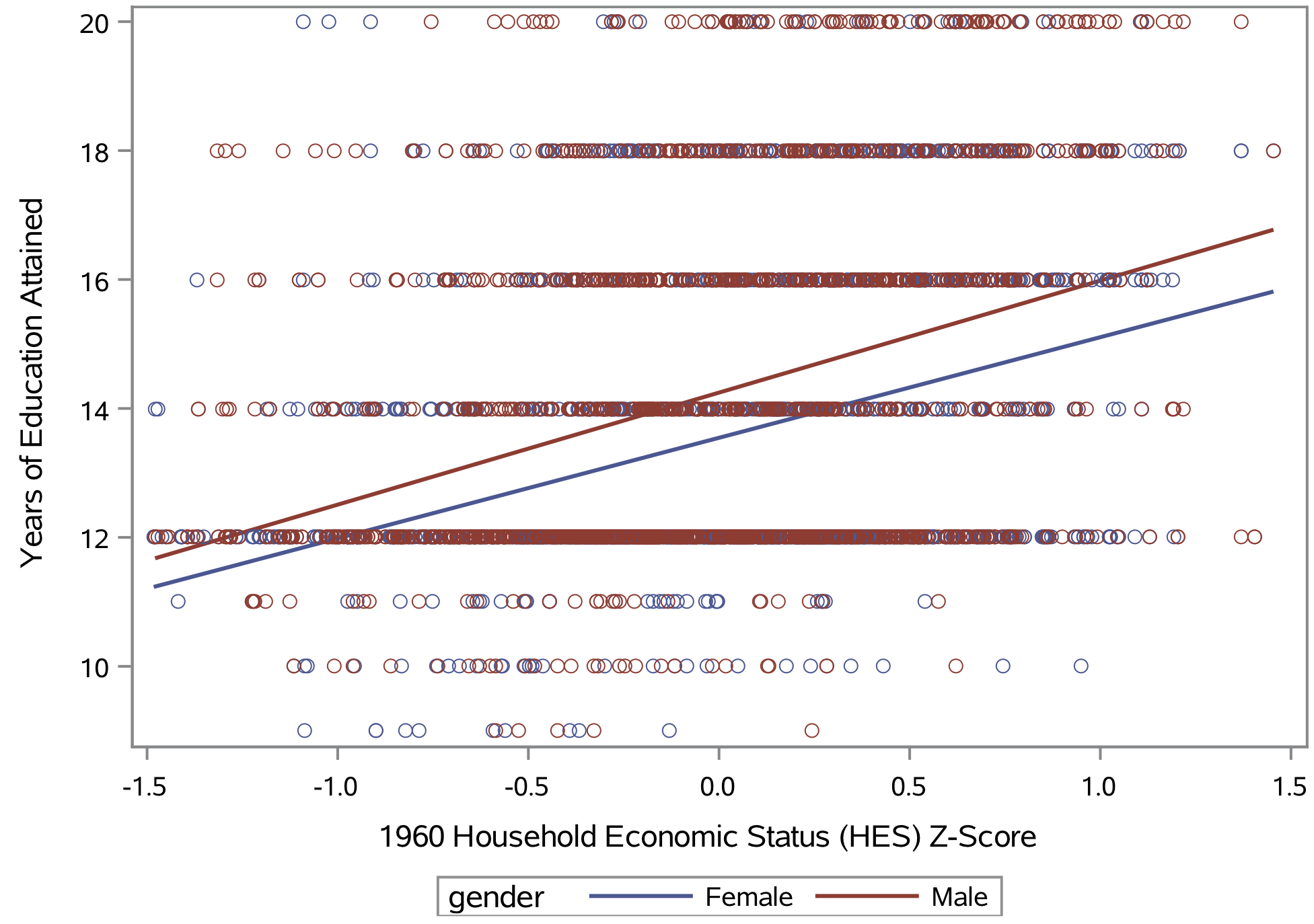


**Supplementary Figure S2**. Effect of the interaction between sex and 1960 family rearing household economic status (HES) on years of education attained

A)
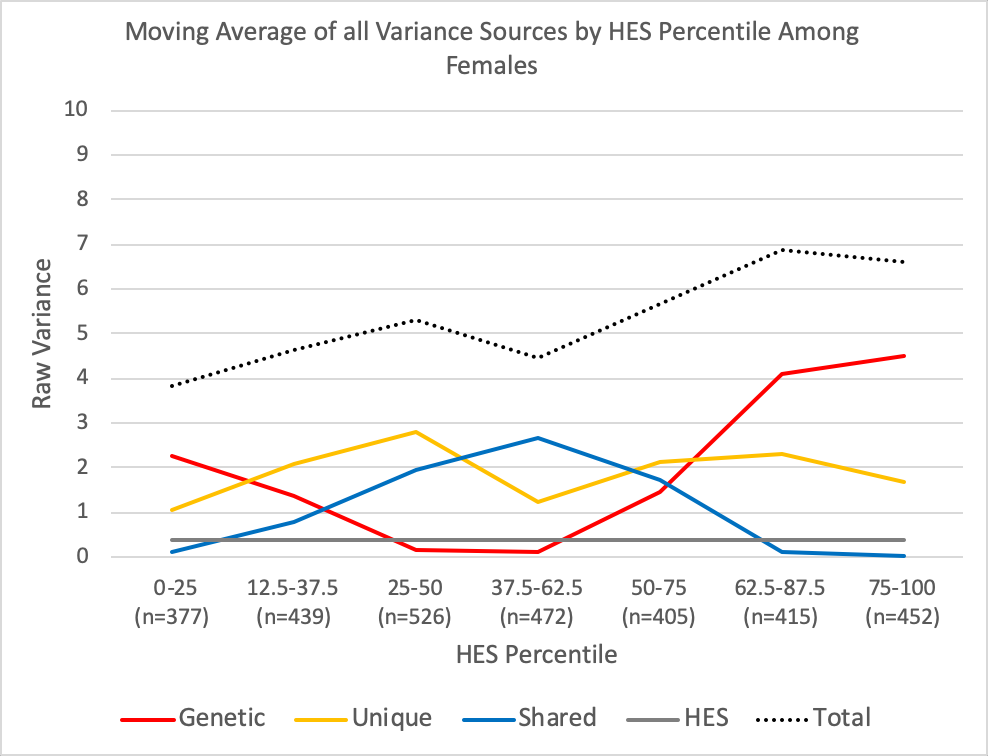
 B)
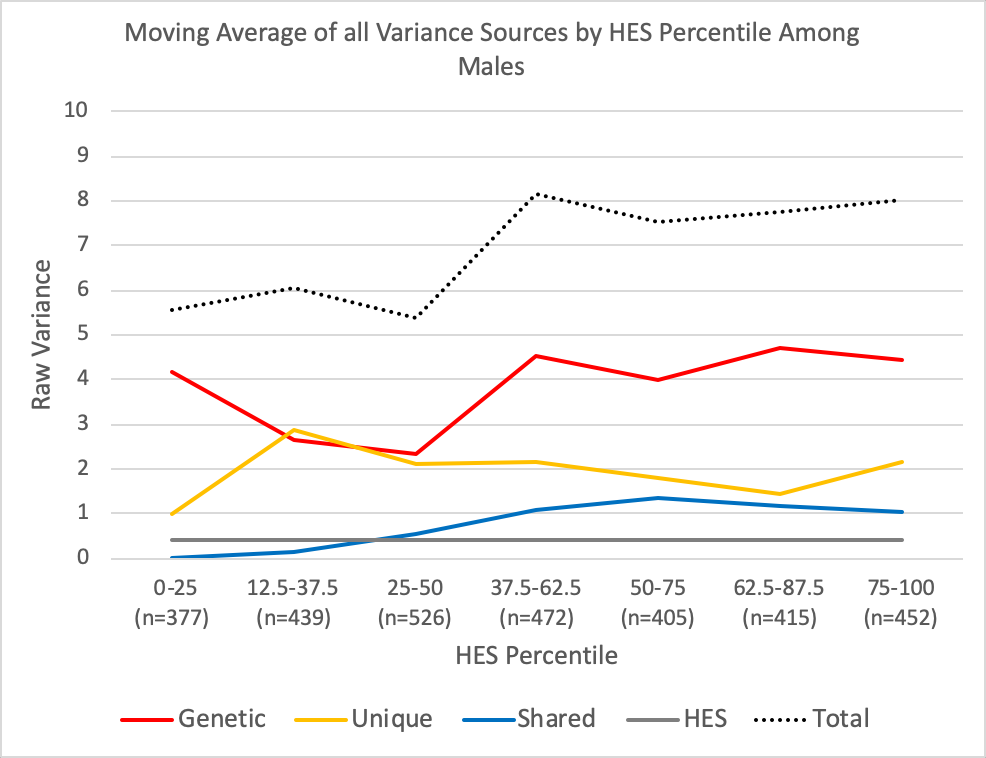


**Supplementary Figure S3.** Moving averages of all variance sources for years of education attained (y-axis), by 1960 family rearing household economic status (HES) percentiles (x-axis), shown separately for (A) women and (B) men. Total variance (black dotted lines) underlying years of education attained increased for both women and men in moving from lowest to highest HES percentile. Genetic variance (red) differed significantly from low to high HES for women but for men remained the same across HES. Genetic variance in highest HES percentile was the same for women and men. For women, Shared Family (blue) and Unique Individual (yellow) variances increase in the middle HES ranges, then is about the same for low and high HES. For men, Shared Family and Unique Individual variances increase in the middle HES ranges, and increase slightly overall from low to high HES. The moving percentile windows show a distinct pattern of changes in genetic variance for EA at HES percentile points of 25% and 75% for women, whereas for men changes were less remarkable across HES percentiles. The pattern shown supports that comparisons across HES can be represented by low, middle and high strata to test for differential contributions of genetic variance.

**Supplementary References**

1. L. Wise, D. McLaughlin, L. Steel, The Project TALENT data handbook, revised. *Palo Alto, CA: American Institutes for Research* (1979).

2. C. A. Prescott *et al.*, The Project Talent Twin and Sibling Study: Zygosity and New Data Collection. *Twin Res Hum Genet* **22**, 769-778 (2019).

3. C. A. Prescott *et al.*, The Project TALENT Twin and Sibling Study. *Twin Res Hum Genet* **16**, 437-448 (2013).

4. A. Kaiser *et al.* (2022) Project Talent Twin and Sibling Study Documentation 2014. (American Institutes for Research, Washington, D.C.).

5. B. Benyamin, I. J. Deary, P. M. Visscher, Precision and bias of a normal finite mixture distribution model to analyze twin data when zygosity is unknown: Simulations and application to IQ phenotypes on a large sample of twin pairs. *Behavior genetics* **36**, 935-946 (2006).

6. L. Christiansen *et al.*, Age- and sex-differences in the validity of questionnaire-based zygosity in twins. *Twin Res* **6**, 275-278 (2003).

7. H. Peeters, S. Van Gestel, R. Vlietinck, C. Derom, R. Derom, Validation of a telephone zygosity questionnaire in twins of known zygosity. *Behav Genet* **28**, 159-163 (1998).

8. E. Spitz *et al.*, Comparative diagnoses of twin zygosity by SSLP variant analysis, questionnaire, and dermatoglyphic analysis. *Behav Genet* **26**, 55-63 (1996).
